# Clofazimine as a substitute for rifampicin improves efficacy of *Mycobacterium avium* pulmonary disease treatment in the hollow-fiber model

**DOI:** 10.1128/aac.01157-23

**Published:** 2024-01-23

**Authors:** Sandra Salillas, Jelmer Raaijmakers, Rob E. Aarnoutse, Elin M. Svensson, Khalid Asouit, Erik van den Hombergh, Lindsey te Brake, Ralf Stemkens, Heiman F. L. Wertheim, Wouter Hoefsloot, Jakko van Ingen

**Affiliations:** 1 Department of Microbiology, Pediatrics, Radiology and Public Health, Faculty of Medicine, University of Zaragoza, Zaragoza, Spain; 2 Radboudumc Community for Infectious Diseases, Department of Medical Microbiology, Radboud University Medical Center, Nijmegen, the Netherlands; 3 Radboudumc Community for Infectious Diseases, Department of Pharmacy, Radboud University Medical Center, Nijmegen, the Netherlands; 4 Department of Pharmacy, Uppsala University, Uppsala, Sweden; 5 Radboudumc Community for Infectious Diseases, Department of Pulmonary Diseases, Radboud University Medical Center, Nijmegen, the Netherlands; St George's, University of London, London, United Kingdom

**Keywords:** nontuberculous mycobacteria, clofazimine, hollow-fiber infection model, pharmacokinetics, pharmacodynamics, PK/PD

## Abstract

*Mycobacterium avium* complex pulmonary disease is treated with an azithromycin, ethambutol, and rifampicin regimen, with limited efficacy. The role of rifampicin is controversial due to inactivity, adverse effects, and drug interactions. Here, we evaluated the efficacy of clofazimine as a substitute for rifampicin in an intracellular hollow-fiber infection model. THP-1 cells, which are monocytes isolated from peripheral blood from an acute monocytic leukemia patient, were infected with *M. avium* ATCC 700898 and exposed to a regimen of azithromycin and ethambutol with either rifampicin or clofazimine. Intrapulmonary pharmacokinetic profiles of azithromycin, ethambutol, and rifampicin were simulated. For clofazimine, a steady-state average concentration was targeted. Drug concentrations and bacterial densities were monitored over 21 days. Exposures to azithromycin and ethambutol were 20%–40% lower than targeted but within clinically observed ranges. Clofazimine exposures were 1.7 times higher than targeted. Until day 7, both regimens were able to maintain stasis. Thereafter, regrowth was observed for the rifampicin-containing regimen, while the clofazimine-containing regimen yielded a 2 Log_10_ colony forming unit (CFU) per mL decrease in bacterial load. The clofazimine regimen also successfully suppressed the emergence of macrolide tolerance. In summary, substitution of rifampicin with clofazimine in the hollow-fiber model improved the antimycobacterial activity of the regimen. Clofazimine-containing regimens merit investigation in clinical trials.

## INTRODUCTION

*Mycobacterium avium* complex pulmonary disease (MAC-PD) is emerging as an important opportunistic infection. The recommended treatment for MAC-PD consists of long-term (18 months) multi-drug therapy combining azithromycin (AZT), ethambutol (EMB), and rifampicin (RIF) with or without (inhaled) amikacin (1). Despite this prolonged multi-drug therapy, outcomes are poor, with only 60%–70% of patients achieving sustained culture conversion (2).

The role of RIF in these treatment regimen is subject of debate since it shows limited activity *in vitro* against MAC bacteria (3), causes frequent and potentially severe adverse events (4), and has pharmacokinetic (PK) interactions with macrolides such as AZT through induction of the CYP3A4 enzyme, resulting in reduced exposure and thus likely reduced efficacy (5, 6).

Replacing RIF with a more effective drug with fewer adverse effects and pharmacokinetic interactions is an important strategy to improve the antibiotic treatment outcome of MAC-PD. Retrospective studies have suggested that clofazimine (CFZ) may be a good alternative to RIF (7, 8) since it is active alone, synergistic with macrolides and amikacin *in vitro* (9), and does not impact AZT exposures *in vivo* (5, 10).

We employed the hollow-fiber infection model (HFIM) to investigate whether CFZ can replace RIF in the recommended treatment of MAC-PD, both in terms of antimycobacterial effect and suppression of the emergence of macrolide tolerance and resistance.

## MATERIALS AND METHODS

### Bacteria, cells, and antibiotics

The *Mycobacterium avium* subsp. *hominissuis* ATCC 700898 reference strain was obtained from the American Type Culture Collection (ATCC; Manassas, VA) and cultured in Middlebrook 7H9 supplemented with 10% oleic albumin dextrose catalase (OADC; Beckton-Dickinson, Vianen, the Netherlands) for 5 days prior to the experiment. THP-1 cells, which are monocytes isolated from peripheral blood from an acute monocytic leukemia patient, were purchased from the German Collection of Microorganisms and Cell Cultures (DSMZ; Braunschweig, Germany; ACC 16 Lot 32) and cultured in RPMI (Roswell Park Memorial Institute medium) 1640 with 20% heat-inactivated fetal bovine serum (FBS; Life Technologies Limited, Paisley, UK) for the first three passages and with 10% FBS afterward at 36°C and 5% CO_2_.

AZT, EMB, RIF, and CFZ were purchased from Sigma Aldrich (Zwijndrecht, the Netherlands). Stock solutions of the compounds were prepared in ethanol, Milli-Q water, dimethyl sulfoxide (DMSO) and DMSO, respectively, and stored at −20°C until use. Syringe solutions of AZT (40%/60% (vol/vol) ethanol/Milli-Q water) and EMB (Milli-Q water) were prepared every 3 days. RIF (in 0.08%/99.2% (vol/vol) DMSO/Milli-Q water) was replaced every 2 days due to drug instability. CFZ bolus solutions at 0.1 mg/mL were prepared daily in 10% DMSO, 0.5% Tween 80 in RPMI-2% FBS.

### Minimum inhibitory concentration (MIC) determinations

To assess the emergence of resistance, the MICs of AZT, RIF, and CFZ against *M. avium* ATCC 700898 were determined prior and post experiment by broth microdilution in cation-adjusted Mueller Hinton broth according to Clinical and Laboratory Standards Institute guidelines (11). For AZT and RIF, microdilution plate concentrations ranged from 256 to 0.125 and from 64 to 0.03 mg/L, respectively. The MIC of CFZ was measured using Sensititre SLOMYCO2 plates (ThermoFisher, Breda, the Netherlands) according to manufacturer’s recommendations.

### Hollow-fiber setup

The intracellular HFIM experiment was performed as previously described (12), but with minor modifications, i.e., a 2·10^6^ cells/mL suspension of THP-1 cells, representing human macrophages, in RPMI 1640 broth containing 2% heat-inactivated FBS was infected with a 0.5 McFarland suspension of *M. avium* and incubated at 36°C in 5% CO_2_ atmosphere for 24 h prior to starting the experiment. Infected THP-1 cells (30 mL) were inoculated into each hollow-fiber cartridge (C8008; FiberCell Systems, New Market, MD, USA) for 4 h before the start of the experiment. All hollow-fiber cartridges were rinsed extensively before use (see Supplement).

Drug penetration into the extracapillary space of the C8008 cartridges was confirmed for AZT, EMB, and RIF (Fig. S1 to S3). CFZ does not penetrate the extracapillary space (Fig. S4) and was administered daily into the extracapillary space via the sampling ports. The cartridges were primed with CFZ before simulating the pharmacokinetic profile to mitigate CFZ’s binding to plastics (Fig. S4).

### Hollow-fiber study design and simulated pharmacokinetic profiles

Three experimental arms were included in triplicate: the rifampicin regimen (AZT, EMB, and RIF), the clofazimine regimen (AZT, EMB, and CFZ), and a growth control.

AZT, EMB, and RIF protein-unbound epithelial lining fluid (ELF) drug concentrations were simulated corresponding to once-daily doses of 250 mg, 900 mg (15 mg/kg), and 600 mg (10 mg/kg), respectively (5, 13–16). We accounted for a 25% increase in AZT exposure when not administered concomitantly with RIF (5). In addition, we considered a [RIF]_ELF_/[RIF]_plasma_ ratio of 2.6, based on available data (15), a free fraction of 10% in plasma and an assumed 100% free fraction in ELF. ELF pharmacokinetic parameters are listed below in Table 1. The diluent inflow was set to simulate the half-life of RIF, and the difference between the half-lives of EMB and AZT was corrected by zero-order top-up.

**TABLE 1:** Pharmacokinetic parameters realized in the HFIM experiment*^a^*

Parameter	Drug	Rifampicin therapy		Clofazimine therapy	
		Target (5, 13–16)	Actual	Target (5, 13–20)	Actual
T_1/2_ (h)	AZT	20	19.2 ± 7.3	20	17.6 ± 1.4
	EMB	10	14.4 ± 5.0	10	10.3 ± 1.5
	RIF	2	3.5 ± 0.7	–	–
T_max_ (h)	AZT	10	15.2 ± 6.0	10	10 ± 0
	EMB	3	3 ± 0	3	2.6 ± 0.6
	RIF	2	2 ± 0	–	–
C_ss_ (mg/L)	AZT	3	4.3 ± 1.2	3.75	4.1 ± 0.2
	EMB	3	2.4 ± 0.1	3	2.6 ± 0.4
	RIF	3.9	2.9 ± 0.3	–	–
AUC_0-24_ (mg·h/L)	AZT	108	75.7 ± 17.2	135	79.1 ± 6.7
	EMB	48.5	39.2 ± 6.1	48.5	32.5 ± 3.3
	RIF	15.2	16.0 ± 2.7	–	–
C_avg_ (mg/L)	CFZ	–	–	2.2	3.7 ± 0.5

^*a*^ Values represent geometric mean ± standard deviation. T_1/2_, elimination half-life; T_max_, time at which C_ss_ is reached; C_ss_, peak concentration at steady state; AUC_0-24_, area under the 24-h concentration-time curve at steady state; C_avg_, average concentration at steady state. Dashes represent PK parameters that were not simulated in each regimen (e.g. RIF PK parameters in the CFZ regimen and vice versa).

An average CFZ concentration at clinical steady state (C_avg_) was targeted at a daily dose of 100 mg. Plasma CFZ concentrations were extrapolated to lung concentrations. A plasma C_avg_ of 0.86 mg/L (17), a [CFZ]_lung_/[CFZ]_plasma_ ratio of 250 (18, 19) and binding to lung tissue similar to that to plasma proteins (99%) (20) were considered, resulting in a C_avg_ of 2.2 mg/L. A CFZ bolus was administered every 24 h. Preparative experiments showed CFZ instability in the HFIM following first-order kinetics with a T_1/2_ degradation time of 14 h (Fig. S4; Table S1). Considering its instability, a C_0_ of 3.7 mg/L was targeted to achieve the desired C_avg_ during the dosing interval.

### Bacterial and THP-1 cell enumerations

Bacterial and THP-1 cell enumeration was performed as previously described (12). Bacterial and THP-1 cell samples were collected on days 0, 3, 7, 14, and 21, and cell densities were determined. Samples were taken directly from the extracapillary space after homogenizing the contents using two 20 mL syringes. A brief overview can also be found in the supplementary materials.

To investigate the emergence of macrolide tolerance, Middlebrook 7H10 agar (M7H10; Beckton-Dickinson, Vianen, the Netherlands) plates were prepared following the manufacturer’s recommendation with the addition of AZT at a final concentration of eight times the pre-treatment MIC. When samples were drawn to determine bacterial density, the samples were also plated on the drug-containing agar plates to detect emergence of macrolide tolerance.

### Pharmacokinetic measurements

Pharmacokinetic evaluation was performed at day 0 of the experiment and at steady state (day 16). For AZT, EMB, and RIF, pharmacokinetic samples were drawn from the central reservoir at timepoints 0, 1, 2, 3, 4, 6, 8, 10, 12, 14, 16, 22, and after 24 h. CFZ samples were drawn 15 minutes after bolus injection, at 12 h and at 24 h from the extracapillary space. Additional CFZ samples were taken simultaneously when samples for bacterial enumeration (pharmacodynamic samples) were drawn from the hollow-fiber cartridge and frozen until further evaluation. Additional samples of CFZ at 1 mg/L were simultaneously frozen as quality controls.

Briefly, all samples were processed by precipitating proteins in the growth medium, followed by a centrifugation step before antibiotic concentrations were analyzed using an ultra-high performance liquid chromatography-mass spectrometer (XEVO TQ-S micro triple quadrupole mass spectrometer, Waters, Etten-Leur, The Netherlands) (see Supplement).

### Calculations and statistics

Pharmacodynamic, THP-1 cell density and pharmacokinetic plots were generated using GraphPad Prism version 7.0.0 (GraphPad Software Inc., LA Jolla, CA, USA). Pharmacokinetic analyses were performed using Phoenix 64 WinNonlin (Build 8.3.1.5.014). The area under the curve (AUC) was calculated using the linear-up log-down trapezoidal rule. The log-linear period (log concentrations versus time) was based on the last data points. The absolute value of the slope (λz /2.303, where λz is the first-order elimination rate constant) was calculated using linear regression analysis. λz allowed the calculation of the antibiotic half-lives (half-life = 0.693/λz). CFZ C_avg_ was calculated by dividing its AUC by 24 h using GraphPad Prism version 7.0.0 (GraphPad Software Inc., LA Jolla, CA, USA). All pharmacokinetic parameters are depicted as the geometric mean of the triplicate values, together with a standard error of the mean.

## RESULTS

### Minimum inhibitory concentrations

The MICs of AZT, EMB, RIF, and CFZ were 64 mg/L, 8 mg/L, 4 mg/L, and 0.12 mg/L, respectively, before the hollow-fiber experiment and remained stable throughout.

### Pharmacodynamic effect and the emergence of macrolide tolerance

**Fig 1:**
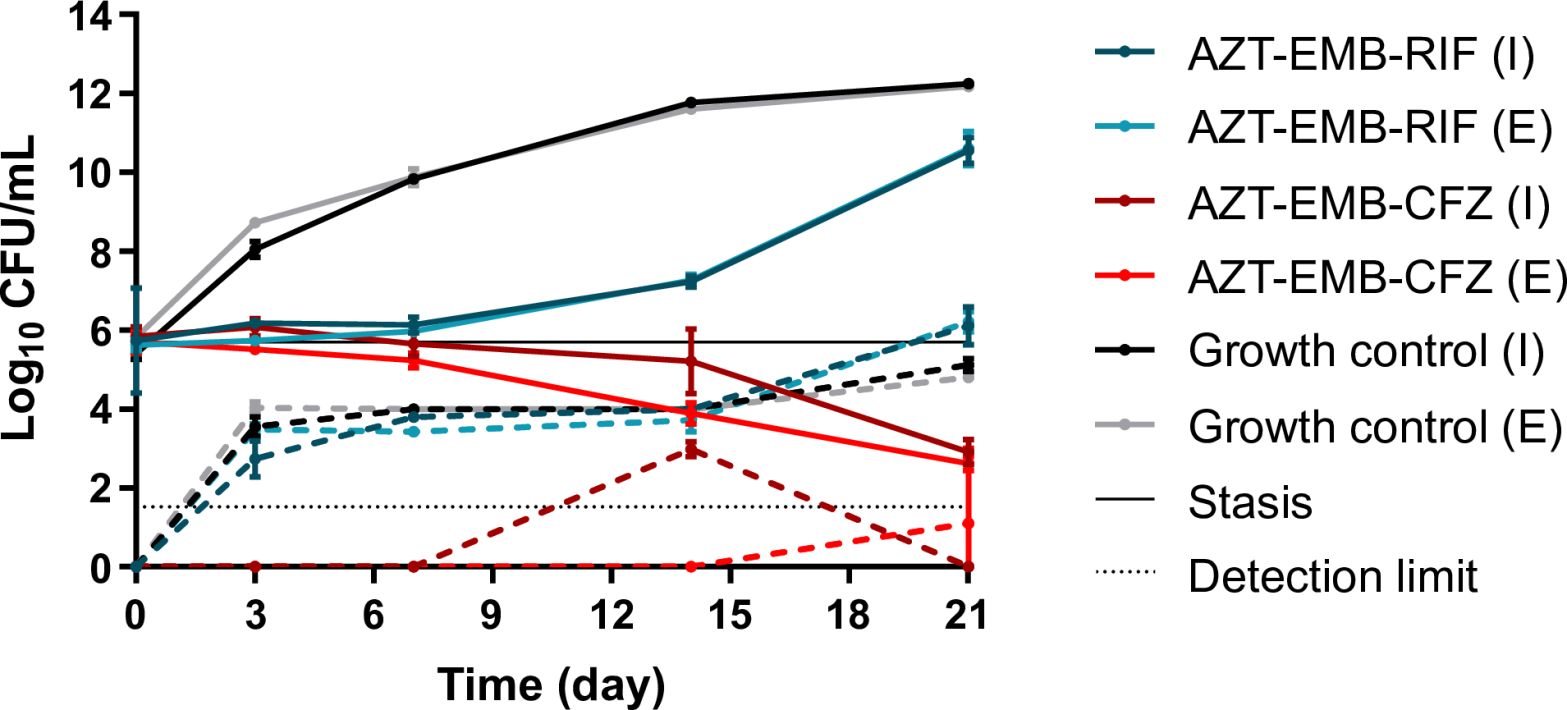
Hollow-fiber pharmacodynamic effects (solid lines) and emergence of macrolide tolerance (dashed lines) of the growth control, rifampicin therapy, and clofazimine therapy in the hollow-fiber experiment. I, intracellular fraction; E, extracellular fraction. Detection limit and stasis correspond to 1.5 Log_10_ CFU/mL and 5.7 Log_10_ CFU/mL, respectively.

Both treatment arms were able to suppress growth of the mycobacterial population until day 7, after which regrowth was observed in the RIF-containing treatment arm (Fig. 1). From day 7, the CFZ-containing regimen was able to reduce the bacterial load by 2 Log_10_ colony forming unit (CFU) per mL at day 21. Specifically, the area under the bacterial kill curve (AUBKC) reduction of the CFZ regimen in comparison to the RIF arm was 35.6%–39.2 % and 22.6%–35.1 % for the extracellular and intracellular fractions, respectively. As seen, the effects on both fractions were similar throughout the experiment. In addition, the CFZ regimen proved to be better at suppressing the emergence of macrolide tolerance (there was an AUBKC reduction between the RIF and CFZ arms of 85%–100% and 72.3%–75.7% for the extracellular and intracellular fractions, respectively), as shown in Fig. 1. On day 7, one growth control system was contaminated and excluded from further analysis.

### Pharmacokinetic evaluation

**Fig 2:**
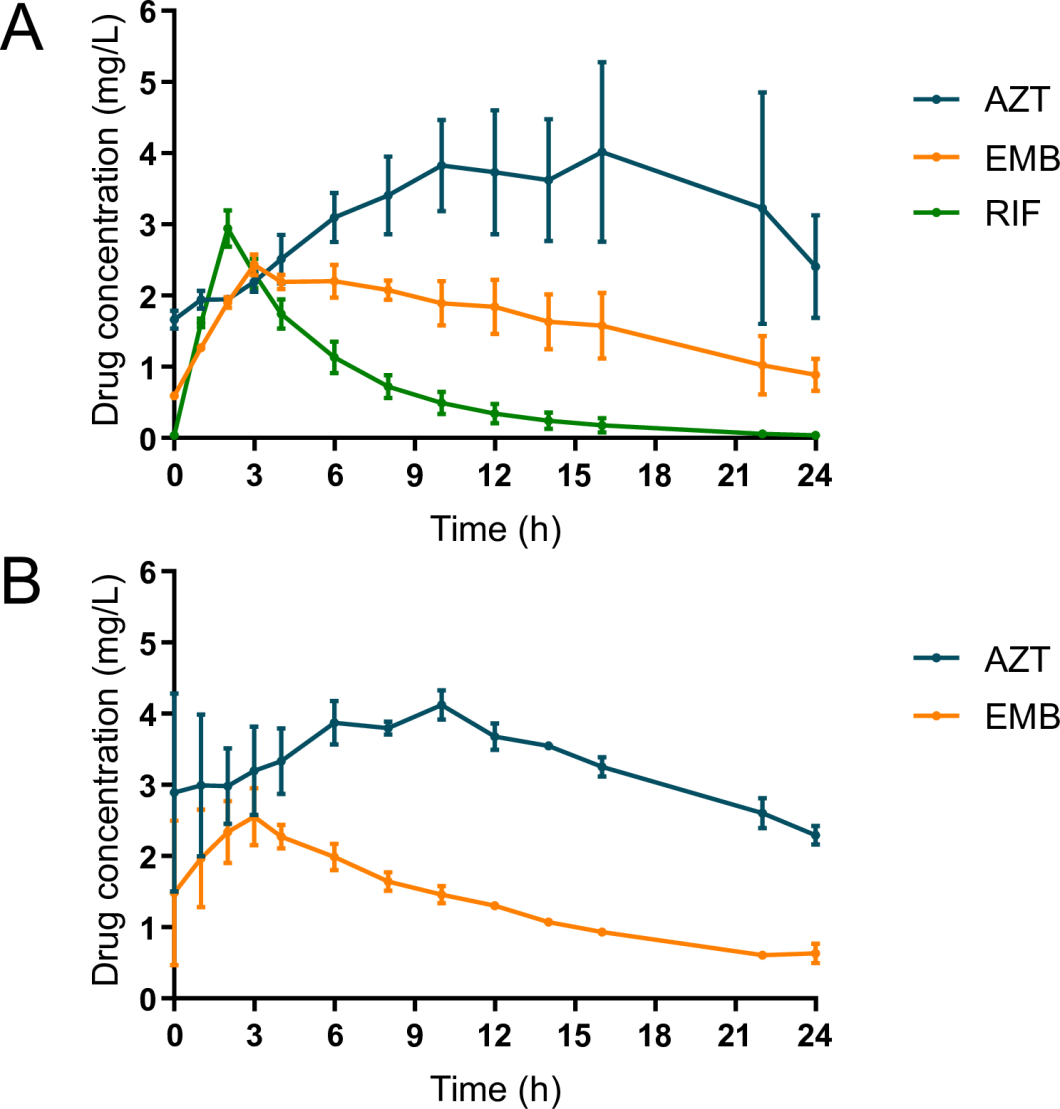
Steady-state pharmacokinetic graph of the rifampicin-containing therapy (**A**) and the clofazimine-containing therapy (**B**) on day 16. Dots represent mean drug concentrations values, which are connected by lines, whereas bars correspond to standard deviations.

Pharmacokinetic parameters and corresponding pharmacokinetic profiles at steady state (at day 16) are shown in Table 1 and Fig. 2. One data point in two systems was not included in the calculation of λz of AZT because of a technical error. The observed AZT exposures were lower than targeted, rendering a 5% increased AZT exposure when it was not administered concomitantly with RIF instead of the 25% targeted. Lower (19%–33%) EMB exposures were also reached, as a result of a low C_max_. RIF exposure followed the targeted profile. The average CFZ concentration was higher than the target (3.8 mg/L instead of 2.2 mg/L).

On day 16, the CultureGuard filter connecting the diluent to one of the CFZ-containing systems was clogged and its PK calculations were excluded from the overall PK analysis.

### THP-1 cells

THP-1 cell density was stable throughout the experiment and between systems (Fig. S5). Due to the vigorous mixing required in the CFZ-containing systems during drug administration, a reduction of THP-1 cells was registered at day 7. Thus, to maintain THP-1 cell counts identical to the other systems, we added 1.7·10^8^ THP-1 cells. The multiplicity of infection started at 1 (bacterium:cells) and increased slightly over time in the growth control and RIF regimens, while the CFZ therapy showed a decrease from day 7 (Fig. S6). It coincided with the decrease in bacterial cells in this arm (Fig. 1). This demonstrates that we were able to maintain the intracellular infection model during the experiment.

## DISCUSSION

Replacing RIF with CFZ improved the antimycobacterial activity of the three-drug macrolide-based regimen in the hollow-fiber model. After an initial phase on par with the RIF-containing regimen, the CFZ-containing regimen showed sustained antimycobacterial activity and suppression of macrolide tolerance and resistance, outperforming the RIF regimen. Given the small differences in AZT exposure between the two regimens (Fig. 2; Table 1), CFZ is the most likely driver of the increased efficacy of the studied regimen. These *in vitro* findings suggest a role for CFZ in MAC-PD treatment regimens and support the inclusion of such a regimen in clinical trials as well as the use of CFZ in patients in whom RIF is contraindicated or intolerable (4, 8).

Biphasic activity, with stasis similar to rifampicin regimen first and bactericidal activity in a later phase, has already been documented for clofazimine. Fourteen days of CFZ monotherapy showed no significant early bactericidal activity in tuberculosis patients (21). Early assessments of CFZ efficacy have not been conducted at MAC-PD, where culture conversion at 6 months of treatment is the first read-out. CFZ-based regimens have shown mixed results there. The recently completed PERC (Pulmonary NTM disease: A regimen of ethambutol and azithromycin with as adjunctive rifampicin vs clofazimine) randomized controlled clinical trial compared CFZ with RIF in the treatment of MAC-PD and recorded equal culture conversion rates (62% vs 58%) after 6 months of treatment (22, 23), similar to the first week in our experiment, while an earlier cohort study suggested higher culture conversion rates for CFZ-containing regimens (8). The late onset of the bactericidal effect of the CFZ regimen is in line with previous observations in mouse models of tuberculosis, where an exposure-dependent bactericidal effect and treatment-shortening potential were observed with prolonged treatment (24). However, recent mouse models of MAC disease showed superior efficacy of CFZ- versus RIF-containing regimens, with early onset of action in both regimens (25).

The effect of CFZ increased equally with time against intracellular and extracellular *M. avium* (Fig. 1), suggesting that intracellular accumulation is not a rate-limiting step. In clinical studies, the late effect is partly due to the very long elimination half-life of CFZ, which consequently takes up to 4 months to reach steady state (26). We simulated steady-state pharmacokinetics yet the full effect was still delayed; in fact, the CFZ exposures in our experiment were higher than targeted, probably due to the mixing process we had to perform after loading CFZ directly into the extracapillary space of the hollow-fiber cartridges, which may better reflect a daily dose of 200 mg. Loading doses, higher doses, or alternative routes of administration such as inhalation (27) may increase and accelerate the effect of CFZ in MAC-PD treatment, but the dose-response relationship of CFZ required to achieve antimicrobial activity remains unknown (24, 26); higher doses up to 600 mg per day and 300 mg per day for 3 months have been used in the treatment of leprosy and leprosy reactions (28, 29). On the other hand, CFZ use has been related to higher mortality rates in the treatment of disseminated MAC infection in patients with AIDS in a single study (30). However, other aspects such as the higher bacterial loads in the CFZ arm may also explain this higher mortality. A study evaluating the safety and pharmacokinetics of a 4-week 300 mg once-daily loading phase of CFZ has recently completed enrolment (clinicaltrials.gov; registration number: NCT05294146). To show the true added benefit of CFZ in the context of MAC-PD, regimens might thus require dose and delivery optimization and benefits might be best shown in longer-term follow-up, e.g., in rates of prolonged culture conversion and relapse rates. Currently, there is very little evidence to support treatment duration in MAC-PD (1) and a potential treatment-shortening effect of CFZ is also an important subject for clinical trials.

RIF lacks *in vitro* activity (3), but it is part of currently recommended treatment regimens for MAC-PD for its presumed potential, when combined with EMB, to prevent the emergence of macrolide resistance (31). Yet, the studies that support this recommendation suggest that EMB is more important than RIF in suppression of macrolide resistance (1, 31), and two recent clinical cohort studies have shown that in low bacterial burden MAC-PD, two-drug regimens of EMB and a macrolide perform as well as the recommended three-drug RIF-containing regimens (32, 33). Our results demonstrate that in the hollow-fiber model, the CFZ regimen is more effective in suppressing macrolide tolerance and resistance. Whether this effect is driven by CFZ or by the higher exposure to AZT cannot be inferred from the current experiment, but the moderate differences in macrolide exposure between the two arms of the experiment and prior studies of CFZ-macrolide synergy (9) suggest that CFZ itself prevents macrolide tolerance.

All limitations inherent to preclinical models also apply to this study. The success of preclinical models in predicting clinical trial outcomes is limited by the assumptions inherent to models. Here, the pharmacokinetics of CFZ in the lung is an important potential limitation. We simulated steady-state pharmacokinetics in the epithelial lining fluid for AZT, EMB, and RIF, but literature on lung concentrations of CFZ is limited and a wide range of drug concentrations in lung tissue and plasma:lung ratios has been reported (18, 19, 34). CFZ pharmacokinetics in ELF have never been reported and our C_avg_ target (0.86 mg/L) was extrapolated from lung tissue concentrations (17). Of note, other authors measured lower concentrations of clofazimine in the lung (about 0.4 mg/L) (5, 26). Moreover, in our experiment, a higher CFZ C_avg_ than targeted was achieved, most likely corresponding to a daily dose of 200 mg instead of 100 mg and this may also be reflected in the positive outcome of the CFZ-containing regimen compared to the RIF regimen. We attained lower AZT exposures than targeted in the CFZ regimen, where targets were higher due to the absence of RIF and its induction of cytochrome P450 enzymes (5). Higher exposures have been shown to improve treatment outcomes in patients (6), so the effect of the CFZ regimen may be underestimated. Finally, our work mainly reflects nodular/bronchiectatic MAC-PD rather than fibro-cavitary disease as we simulate ELF pharmacokinetics and this does not reflect the lower exposures observed in cavity walls and contents (35).

In conclusion, replacing RIF with CFZ improved the efficacy of MAC-PD treatment in the hollow-fiber model. The CFZ concentrations we achieved are steady-state concentrations and may better correspond to a daily dose of 200 mg instead of 100 mg. Therefore, higher doses, the safety of higher loading doses, and targeted delivery of high doses should be investigated in human subjects. Findings from this study bring hope for an improved regimen but should be confirmed in clinical trials.

## SUPPLEMENTAL MATERIAL

Supplemental material aac.01157-23-s0001.docx Supplemental text, figures, and tables.

ASM does not own the copyrights to Supplemental Material that may be linked to, or accessed through, an article. The authors have granted ASM a non-exclusive, world-wide license to publish the Supplemental Material files. Please contact the corresponding author directly for reuse.
