# Supplementary material for: Clofazimine as a substitute for rifampicin improves efficacy of Mycobacterium avium pulmonary disease treatment in the hollow-fiber model
Source: Antimicrob Agents Chemother. 2024 Jan 23;68(3):e01157-23. doi: 10.1128/aac.01157-23 (PMC10916390; doi:10.1128/aac.01157-23)
Supplement: Supplemental material — Supplemental text, figures, and tables. [file aac.01157-23-s0001.docx]

Supplementary Material

1. Penetration of azithromycin, ethambutol and rifampicin to the extra capillary space of the cartridge
2. Validation experiment of the use of clofazimine in the hollow-fibre infection model
3. THP-1 cell densities and multiplicity of infection
4. Preparation of the cartridges and the hollow-fibre system
5. Pharmacodynamic and pharmacokinetic samples processing and measurement
6. Unexpected events during the experiment
7. References
8. **Penetration of azithromycin, ethambutol and rifampicin to the extra capillary space of the cartridge**

Figure S1: Azithromycin (AZT) can penetrate the cellulose fibres of the C8008 cartridge because after administration of the drug into the central reservoir, its concentration there and in the extra capillary space is quite similar.

Figure S2: Ethambutol (EMB) can penetrate the cellulose fibres of the C8008 cartridge, because after administration of the drug into the central reservoir, its concentration there and in the extra capillary space is quite similar.

Figure S3: Rifampicin (RIF) can penetrate the cellulose fibres of the C8008 cartridge because after administration of the drug into the central reservoir, its concentration there and in the extra capillary space is quite similar.

1. **Validation experiment of the use of clofazimine in the hollow-fibre infection model**

2.1. Methods

The hollow-fibre setup we used to perform this experiment is the same as that described in the main text of the manuscript.

The pharmacokinetic properties we simulated in this validation experiment were based on the average steady state concentration (C_avg_) of clofazimine (CFZ) after administration of 100 mg/day to MAC-PD patients. To calculate it, we followed the same strategy that is described in the “hollow-fibre study design and simulated pharmacokinetic profiles” section of the paper and considered the same protein binding and [CFZ]_lung_/[CFZ]_plasma_ ratio. The only difference is that here we considered a degradation half-life of clofazimine of 23 hours instead of 14 hours as we had obtained results in previous experiments (data not shown). In summary, the PK parameters we targeted were:

| **Drug** | **Clofazimine** |
| --- | --- |
| Simulated dose | 100 mg/day |
| C_avg_ | 2.15 mg/L |
| C_0_ | 2.98 mg/L |
| T_max_ | 0 h (bolus) |
| T_1/2_ | 23 h (degradation of CFZ in the HFS) |

C_avg_: average concentration of clofazimine at steady state; C_0_: initial concentration of clofazimine to achieve the corresponding C_avg_; T_max_: time at which C_max_ is reached; T_1/2_: half-life.

2.2. Sample preparation

1.  Mix the cartridges using two 20 mL syringes and take a 0.5 mL sample from the cartridge (extra capillary space)

2.  Spin down 0.4 mL from each sample for 10 min at 1500 rpm

3.  The supernatant was removed to measure the CFZ extracellular concentration

4.  The pellet was dissolved in 0.4 mL of water + 0.05 % tween 80 for THP-1 cell lysis and extract the intracellular clofazimine

5.  Thaw and mix the (pre) created calibrators and quality controls (qc)

6.  In safe-lock tubes, dilute the calibrators and qc a factor 10 in RPMI 1640+2% FBS (e.g. 40µL in 360 µL RPMI 1640+2% FBS)

7.   Mix the diluted calibrators and qc with a multi-tube vortex

8.  Pipette in a safe-lock tube: 50 µL sample (intra/extracellular fraction), diluted qc or calibrator and add 150 µL protein precipitation (PP) solution

9.   Mix with a multi-tube vortex 20 sec. speed 2500 rpm

10. Centrifuge 5 min (18.620 g)

11.  Add to the autosampler vials: 95 µL formic acid 1 mg/mL and 75 µL supernatant

12. Close the autosampler vial with a pre-slit cap

14. Mix the autosampler vials with a multi-tube vortex 20 sec. speed 2500 rpm

15. Inject the sample(s) on the chromatographic separation system

 2.3. Results

By administering clofazimine directly into the extra capillary space of the HF cartridge, we were able to simulate a PK profile for clofazimine. We were also able to detect the drug inside the THP-1 cells, which means that clofazimine was able to cross their plasmatic membrane and enter the intracellular space (Figure S4).

If we focus on the extracellular concentration of clofazimine where the PK curves are clearer (Figure S4), we see that the exposure of clofazimine is slightly lower than targeted, probably due to the faster degradation of the drug than anticipated (shorter half-life: 14 h vs 23 h) (Table S1).

With this experiment, we were able to show that we could simulate the PK profile of clofazimine and detect not only the extracellular, but also the intracellular concentration of clofazimine (within the THP-1 cells used to infect *M. avium*). It also allowed us to determine the degradation of clofazimine within the HFS, which followed first-order kinetics. To determine it, we calculated the average of the half-life of the curves we obtained on days 2 and 3 (Figure S4 and Table S1) as the shape was more realistic, and the half-life obtained was 14 hours.

This experiment also demonstrated that clofazimine could not overcome the cellulose fibres of the cartridge, as no clofazimine was detected in the central reservoir after the drug was administered directly into the extra capillary space of the cartridge (Figure S4). The same repulsion effect was observed when polysulfone (C2011) and PVDF (C7011) cartridges were used (data not shown).

Figure S4: Clofazimine concentration over time. Clofazimine was administered at time 0 every day for 3 days and its extra and intracellular concentration in the extra capillary space (ES) of the cartridge was measured at 0.017, 2, 6, 12 and 24 hours after administration. The amount of CFZ we administered on day 1 and 2 was mistakenly the same even when we wanted to administer a lower amount of the drug from day 2 onwards to simulate the same exposure over the course of the experiment. On day 3, a lower amount of CFZ was administered to maintain the AUC of the drug over time. Samples were also taken from the central compartment (CR) of the cartridge to demonstrate that the drug was unable to pass through the fibres of the cartridge.

Table S1: Pharmacokinetic parameters obtained for clofazimine in the validation experiment

| **[CFZ]_extracellular fraction_** | | | | | |
| --- | --- | --- | --- | --- | --- |
|  | C_0_ (mg/L) | T_max_ (h) | T_1/2_ (h) | AUC_0-24h_ (mg·h/L) | C_avg_ (mg/L) |
| Day 1 | 3.52 | 0.02 | 15.34 | 36.71 | 1.53 |
| Day 2 | 5.09 | 0.02 | 13.30 | 37.76 | 1.57 |
| Day 3 | 3.03 | 0.02 | 14.75 | 27.57 | 1.15 |
| **[CFZ]_intracellular fraction_** | | | | | |
|  | C_0_ (mg/L) | T_max_ (h) | T_1/2_ (h) | AUC_0-24h_ (mg·h/L) | C_avg_ (mg/L) |
| Day 1 | 2.99 | 2 | 18.86 | 42.44 | 1.76 |
| Day 2 | 1.91 | 0.02 | 13.30 | 22.86 | 0.95 |
| Day 3 | 1.79 | 0.02 | 10.08 | 10.76 | 0.45 |

C_0_: initial concentration of clofazimine to achieve the corresponding C_avg_; T_max_: time at which C_max_ is reached; T_1/2_: degradation half-life; AUC_0-24_: area under the concentration-time curve from 0 to 24 h; C_avg_: average concentration of clofazimine at steady-state.

1. **THP-1 cell densities and multiplicity of infection**

Figure S5: THP-1 cell concentrations over time. THP-1 cell density was stable and did not show much variation among the systems. This allowed us to maintain an intracellular model throughout the experiment.

Figure S6: Multiplicity of infection (MOI) over time. The MOI started at about 1 bacterium per eukaryotic cell and increased slightly over time for the growth control and standard regimen, while the clofazimine therapy showed a decrease from day 7, corresponding to the killing of the bacteria by this regimen.

1. **Preparation of the cartridges and the hollow-fibre system**

4.1. Preparation of the cartridges

Before the experiment, all cartridges were washed with milli-Q water in a closed circuit with a total volume of 370 mL (which corresponds to the volume of the cartridge (50 mL), tubing (20 mL) and central reservoir (300 mL)). We then connected the medium and waste bottles to the systems and washed them with miliQ water for 24 h and, then with RPMI-2%FBS for additional 24 h.

Saturation of the cartridges with clofazimine was done on days -4 to -2. To do this, we prepared a fresh 0.1 mg/mL clofazimine solution in 10% DMSO, 0.5% Tween 80 in RPMI-2% FBS and added 2 mL/system three times daily by using a 3 mL syringe (Beckton-Dickinson, Vianen, the Netherland). On day -1, these cartridges were washed with 1 L RPMI to remove the excess of unbound clofazimine.

4.2. Hollow-fibre system

Diluent medium, consisting of RPMI1640 with 2% FBS, was pre-filtered with autoclaved Culture Guard 0.22 µm filters (Repligen, Waltham, MA, USA) and pumped into the system with peristaltic pumps (530S with 313x pump head extensions, Watson Marlow, Barendrecht, the Netherlands) at a rate of 2.1 mL/min. Tubing to and from the system was Pumpsil tubing with 1.6 mm Bore x 1.6 mm wall thickness made of platinum-cured silicone tubing (Watson Marlow, Barendrecht, NL) fitted with 3/32 ID luer fittings.

4.3. Clofazimine administration to the hollow-fiber cartridges

CFZ bolus solutions at 0.1 mg/mL were prepared daily in 10% DMSO, 0.5% Tween 80 in RPMI-2% FBS, being the final concentration of DMSO in the system (0.26-0.37%) lower than that found harmful for THP-1 cells (1%) (data not shown).

1. **Pharmacodynamic and pharmacokinetic samples processing and measurement**

5.1. Bacterial enumeration

Before sampling, the cartridges were mixed vigorously using two 20 mL syringes, and 2 mL of suspension was drawn from the cartridge. 1 mL of this sample was spun at 1500 rpm for 10 min, and the supernatant was removed for extracellular bacterial enumeration.

From each sample, 20 µL was taken and mixed 1:1 with a trypan blue solution (Sigma-Aldrich, Zwijndrecht, The Netherlands) to determine the concentration of THP-1 cells in the sample. 10 µL of the mixture was inserted in plastic counting slides (KOVA International, CA, USA) before counting the THP-1 cells using a microscope (10x lens).

For bacterial enumeration, 1 mL of the sample was transferred to a 15 mL sterile Greiner tube and spun at 1500 rpm for 10 minutes. The supernatant was transferred to a new Greiner tube to determine the extracellular bacterial fraction. The pellet was resuspended in 1 mL Milli-Q water + 0.05% Tween 80 to lyse the THP-1 cells and then determine the intracellular bacterial fraction. Middlebrook 7H10 (M7H10) plates were prepared according to manufacturers' recommendations (Beckton-Dickinson, Vianen, the Netherlands). Each sample was 10-fold serial diluted in saline + 10% bovine serum albumin to avoid carry-over of clofazimine (1, 2) and plated on the M7H10 plates in 3 drops of 10 µL each. The plates were incubated at 36 ºC for 7 days. After the bacteria had grown, the colonies were counted and the initial concentrations of the bacteria were calculated.

The multiplicity of infection (MOI) was determined for the intracellular fraction of the bacteria and plotted against time.

5.2. Pharmacokinetic measurements

Time points were chosen to record both peak drug concentrations as well as trough levels in order to verify that the pharmacokinetic parameters were simulated correctly. We first flushed the filtered clave connector by removing 1 mL of medium through it, and then took a 1 mL sample, ensuring only fresh medium was collected. The samples were processed immediately.

5.2.1. Calculations

Pump rates in both drug syringes and media pumps were calculated using standard pharmacokinetic equations. To mimic the half-lives of the different drugs, the pumps were set to accurately eliminate the drug with the shortest half-life. To achieve different other drug half-lives, we then calculated how much additional drug would need to be injected per time unit of the other drugs to artificially prolong their apparent half-lives. The elimination rate constant k was calculated with k = ln(2)/t_1/2_, where t_1/2_ is the half-life of the drug with the shortest *in vivo* half-life. The clearance (CL) per hour was calculated using CL = k * V_D_, where V_D_ was the total volume of the system.  During infusion, we calculated the drug concentration (C) using C = k_0_/CL * (1- e^-k*t^), where k_0_ is the infusion rate and k is the elimination rate constant calculated previously.  The concentration of drug at time t after infusion was calculated using C_t_ = C_0_ * e^-kt^ where C_0_ is the concentration at T_max_ and k is the elimination rate constant.  We assumed that the drug concentration is no longer detectable after 5 x half-lives.

Table S2: Syringe pump settings for daily infusions

| Rifampicin Regimen | | | |
| --- | --- | --- | --- |
| Drug | Infusion Volume (mL/h) | Time (hh:mm) | Syringe concentration (mg/L) |
| Azithromycin | 0.99276 | 10:00 | 400 |
|  | 0.79998 | 05:15 | 400 |
|  | 0.66 | 04:30 | 400 |
|  | 0.58002 | 04:15 | 400 |
| Ethambutol | 1.98366 | 03:00 | 300 |
|  | 0.9 | 04:45 | 300 |
|  | 0.63336 | 04:15 | 300 |
|  | 0.43332 | 06:30 | 300 |
|  | 0.28332 | 05:30 | 300 |
| Rifampicin | 12.18206 | 02:00 | 80 |
|  |  |  |  |
| Clofazimine Regimen | | | |
| Azithromycin | 0.99276 | 10:00 | 500 |
|  | 0.79998 | 05:15 | 500 |
|  | 0.66 | 04:30 | 500 |
|  | 0.58002 | 04:15 | 500 |
| Ethambutol | 1.98366 | 03:00 | 300 |
|  | 0.9 | 04:45 | 300 |
|  | 0.63336 | 04:15 | 300 |
|  | 0.43332 | 06:30 | 300 |
|  | 0.28332 | 05:30 | 300 |
| Clofazimine | First day | 1.125 mL (100 mg/L) | Solvent:  10% DMSO  0.5% Tween 80  89.5% RPMI-2% FBS |
|  | Other days | 0.846 mL (100 mg/L) |  |
| System parameters | | | |
| Pump inflow | 2.1 mL/min | Distribution Volume | 370 mL |

5.2.2. Drug concentration measurement

Briefly, after precipitating the samples with acetonitrile or acetonitrile/methanol 75/25 % (v/v), they were injected on a Xbridge C18 3.5 µm 2.1x50 mm column in the case of azithromycin or an Acquity UPLC BEH C18 1.7 µm 2.1x100 mm for ethambutol, rifampicin and clofazimine. For all drugs, the mobile phase consisted of water and acetonitrile at a concentration of 95/5 % (v/v) supplemented with 0.1 % formic acid. Calibration curves (at 0.3-30 mg/L for azithromycin, 0.5-150 mg/L for ethambutol, 1.6-500 mg/L for rifampicin and 0.04-5 mg/L for clofazimine) and three or four quality controls (at low, medium, high and extra-high concentrations) were run in duplicate in all assays. If we expected the concentration of a sample to be out of range, we diluted it in RPMI-2% FBS before processing. The transitions from precursor drugs to product ions were 749.5 to 116.0 m/z, 205.0 to 116.0 m/z, 823.2 to 791.4 m/z and 473.16 to 431.04 m/z for azithromycin, ethambutol, rifampicin and clofazimine, respectively. Azithromycin [^13^C, ^2^H_3_], ethambutol ^2^H_4_, rifampicin ^2^H_8_ and clofazimine ^2^H_6_ were included as internal standards. Only runs in which the samples from the calibration curves and quality controls did not exceed ±15% of their theoretical concentration were accepted.

5.2.3. Clofazimine drug profile characterization

Figure S7: Steady-state pharmacokinetic graph of the clofazimine replaced therapy on day 16

Figure S8: Clofazimine concentration along the experiment on the clofazimine-containing systems

5.2.4. Pharmacokinetic Measurement protocols

5.2.4.1. Azithromycin Pharmacokinetic Sample measurement protocol

Reference materials

- Azithromycin European Pharmacopoeia (EP) Reference Standard (Sigma-Aldrich, Zwijndrecht, The Netherlands)
- Azithromycin [^13^C, ^2^H_3_] (Alsachim, Illkirch-Graffenstaden, France)

Chemicals and solvents

- Acetonitrile, Hypergrade for LC-MS (Merck, Darmstadt, Germany)
- Methanol, Absolute ULC/MS (Biosolve, Valkenswaard, The Netherlands)
- Formic acid, 98-100% for LC-MS (Merck, Darmstadt, Germany)
- RPMI Medium 1640 (Thermo Fisher Scientific, Breda, The Netherlands)
- Fetal bovine serum (Thermo Fisher Scientific, Breda, The Netherlands)

 Stocks and work solutions

For azithromycin, three independent stock solutions (for calibrators, quality controls, and a reserve) were prepared at a concentration of 1 mg/mL and stored at -40 °C. AZT stocks were dissolved with methanol. The labelled internal standard stock is prepared and stored in the same manner as the unlabelled compound stocks, at 1 mg/mL. From the labelled internal stock solutions, a protein precipitation solution is made with a concentration of 0.1 mg/L in acetonitrile and stored at -40°C.

Calibration and quality control solutions

For the preparation of the calibration curve, one of the stock solutions was used and diluted with acetonitrile to achieve seven calibration solutions 0.30–0.54–1.95–3.75–7.5–15-30 mg/L AZT.

Quality control solutions are made at three levels (low, medium and high) from a different stock than the calibration solutions containing 0,4-6-20 mg/L for AZT. During sample preparation, these working solutions are further diluted (factor 20) in RPMI 1640+2%. All solutions were stored at −80 °C until analysis and are stable for at least 7 months.

Equipment and settings

Table S3:  Pump settings used for ACQUITY UPLC H-Class QSM for azithromycin determinations

| **Pump settings (ACQUITY UPLC H-Class QSM; Waters, Milford, MA, USA)** | | | |
| --- | --- | --- | --- |
| **Time (min)** | **Flow rate (mL/min)** | **% A**  Water + 0.1% formic acid | **% B**  Acetonitrile + 0.1% formic acid |
| 0 | 0.3 | 98 | 2 |
| 1.00 | 0.3 | 30 | 70 |
| 2.00 | 0.6 | 30 | 70 |
| 3.00 | 0.1 | 30 | 2 |
| 3.25 | 0.3 | 98 | 2 |
| 5.50 | 0.3 | 98 | 2 |
| 6.00 | 0.3 | 98 | 2 |
|  | | | |
| Seal wash: 90/10 % (v/v) water/acetonitrile | | | |
| Seal wash period: 5 min | | | |

Table S4:  Autosampler and column oven settings for azithromycin pharmacokinetic determinations

| **Autosampler and column oven (ACQUITY UPLC H-Class SM-FTN; Waters, Milford, MA, USA)** |
| --- |
| Purge solvent: 95/5 % (v/v) water/acetonitrile + 0.1% formic acid |
| Wash solvent: 40/60 % (v/v) water/acetonitrile + 1% formic acid |
| Injection volume: 1 µL |
| Pre-inject wash time: 5 sec |
| Post-inject wash time: 5 sec |
| Needle placement: 4 mm |
| Column Temperature 25 ±1°C |
| Column: Xbridge C18 3.5 µm 2.1x50 mm |
| Temp. tray: 10°C |

Table S5: Ionspray source used for azithromycin pharmacokinetic determinations

| **Ionspray source** |
| --- |
| Capillary: 2 kV |
| Polarity positive ion mode |
| Desolvation temperature: 500 °C |
| Desolvation gas flow: 950 L/h |
| Cone flow: 10 L/h |

Table S6: Mass spectrometer settings used for azithromycin pharmacokinetic determinations

| **MS settings (XEVO TQS-micro; Waters, Milford, MA, USA)** | | | | | |
| --- | --- | --- | --- | --- | --- |
| **Compound Name** | **Parent (m/z)** | **Daughter (m/z)** | **Dwell (s)** | **Cone (V)** | **Collision (V)** |
| Azithromycin | 749.5 | 116.0 | 0.1 | 16 | 48 |
| Azithromycin [^13^C, ^2^H_3_] | 753.4 | 158.1 | 0.1 | 46 | 36 |
|  | | | | | |
| **Ionspray source** | | | | | |
| Capillary: 2 kV | | | | | |
| Polarity: positive ion mode | | | | | |
| Desolvation temperature: 500 °C | | | | | |
| Desolvation gas flow: 950 L/h | | | | | |
| Cone flow: 10 L/h | | | | | |

Accessories and disposables

Table S7: Accessories and disposable used for AZT pharmacokinetic determinations

| **Name** |
| --- |
| Finnpipette® Air displacement pipette 50-200 µL (ThermoFisher Scientific, Breda, The Netherlands) |
| Finnpipette® Air displacement pipette 5 - 40 µL (ThermoFisher Scientific, Breda, The Netherlands) |
| Finnpipette® Pipette tips 250 µL (ThermoFisher Scientific, Breda, The Netherlands) |
| HandyStep ® Repeater pipette (Brand, Wertheim, Germany) |
| HandyStep ® Repeater pipette tip 5 mL (Brand, Wertheim, Germany) |
| Autosampler vials: TPX micro-vial ND9 insert int. 0.2 mL TPX clair 32x11.6mm (VWR, Amsterdam, The Netherlands) |
| Autosampler caps: PP Screwcap 9 mm pre-slit septum (VWR, Amsterdam, The Netherlands) |
| Safe-Lock tube 1.5 mL (Eppendorf, Nijmegen, The Netherlands) |
| DVX-2500 Multi-Tube Vortexer (VWR, Amsterdam, The Netherlands) |

Sample preparation

1.  Thaw and mix the (pre) created calibrators and qc

2.  In safe-lock tubes, dilute the calibrators and qc a factor 20 in RPMI 1640+2% FBS (e.g. 30 µL in 570 µLRPMI 1640+2% FBS)

3.  Mix the diluted calibrators and qc with a multi-tube vortex

4.  Pipette in a safe-lock tube: 50 µL sample, diluted qc or calibrator and add 100 µL PP solution

5.  Mix with a multi-tube vortex 20 sec. speed 2500 rpm

6.  Centrifuge 5 min (18.620 g)

7. Add 120 µL supernatant to numbered 200 µL HPLC vials

8.  Close the autosampler vial with a pre-slit cap

9.  Inject the sample(s) on the chromatographic separation system

Calculations

Acquired data were processed using Waters TargetLynx software (version 4.1). Calibration curves were fitted linearly as the ratio of the peak area of the compound signal response and the peak area of the internal standard against concentration, and a weighting factor of 1/x2 was also used.

Chromatogram


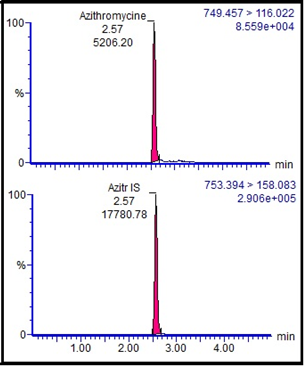


 Figure S9: Chromatogram of the lowest calibrator with internal standard

 Validation results

Table S8: Within – and between run accuracy and precision for azithromycin

|  | Conc. | Within run (n=5) | | Between run (n=15) | |
| --- | --- | --- | --- | --- | --- |
|  | (mg/l) | Accuracy (%) | Precision (%) | Accuracy (%) | Precision (%) |
| AZT | 0.0150  0.0190  0.301  1.004  1.507 | 97.9  91.8  94.5  93.4  95.9 | 8.69  4.42  5.43  7.61  8.12 | 99.1  94.3  98.1  97.6  98.5 | 8.69  3.88  3.36  2.85  8.12 |

5.2.4.2. Ethambutol and rifampicin pharmacokinetic sample measurement protocol

Reference materials

- Ethambutol dihydrochloride salt, antimycobacterial (Sigma-Aldrich, Zwijndrecht, The Netherlands)
- Ethambutol ^2^H_4_ (Alsachim, Illkirch-Graffenstaden, France)
- Rifampicine >97% powder (Sigma-Aldrich, Zwijndrecht, The Netherlands)
- Rifampicine ^2^H_8_ (Alsachim, Illkirch-Graffenstaden, France)

Chemicals and solvents

- Acetonitrile, Hypergrade for LC-MS (Merck, Darmstadt, Germany)
- Methanol, Absolute ULC/MS (Biosolve, Valkenswaard, The Netherlands)
- Ascorbic acid, Ph.Eur. (Spruyt-Hillen, IJselstein, The Netherlands)
- Formic acid, 98-100% for LC-MS (Merck, Darmstadt, Germany)
- Ammonium formate, Eluent additive for LC-MS (Sigma Aldrich, Zwijndrecht, The Netherlands)
- RPMI Medium 1640 (Thermo Fisher Scientific, Breda, The Netherlands)
- Fetal bovine serum (Thermo Fisher Scientific, Breda, NL)

Stock and work solutions

Three independent stock solutions (for calibrators, quality controls and a reserve), were prepared and stored at -40 °C. Ethambutol stock solution (1 mg/mL) was prepared in water and rifampicin stock solution (2 mg/mL) in methanol/water 80/20 % (v/v) with 0.16 mg/mL ascorbic acid.

The labelled internal standard stocks are prepared by dissolving the compounds in the same manner as the unlabelled compounds stocks, only at 1 mg/mL.

A protein precipitation solution containing 0.1 mg/L EMB and 0.1 mg/L RIF in acetonitrile/methanol 75/25 % (v/v) is prepared from the labelled internal stock solutions. All stocks and solutions were stored at -40°C.

Calibration and quality control solutions

For the preparation of the calibration curve, one of the stock solutions was used and diluted with methanol/water 50/50 % (v/v) + 0.16 mg/mL ascorbic acid to obtain seven calibration solutions 0.50–0.75–4.0–11–38–75–150 mg/L EMB and 1.6–2.5–12.5–38–125–250–500 mg/L RIF.

Quality control samples are made at three levels (qc low, medium and high) from a different stock than the calibration solutions with 1.1–11–120 mg/L EMB and 4–40–400 mg/L RIF.

All solutions were stored at −40 °C until analysis and are stable for at least 6 months.

Equipment and settings

Table S9: Pump settings used for ACQUITY UPLC H-Class QSM for rifampicin and ethambutol pharmacokinetic determinations

| **Pump settings (ACQUITY UPLC H-Class QSM; Waters, Milford, MA, USA)** | | | |
| --- | --- | --- | --- |
| **Time (min)** | **Flowrate (mL/min)** | **% A**  10 mM ammonium formate buffer in water pH 4.5 | **% B**  10 mM ammonium formate buffer in methanol pH 4.5 |
| 0 | 0.3 | 98 | 2 |
| 2.05 | 0.3 | 70 | 30 |
| 4.00 | 0.3 | 10 | 90 |
| 5.00 | 0.3 | 10 | 90 |
| 5.10 | 0.3 | 98 | 2 |
| 10.00 | 0.3 | 98 | 2 |
|  | | | |
| Seal wash: 90/10 % (v/v) water/acetonitrile | | | |
| Seal wash period: 5 min | | | |

Table S10: Autosampler and column oven settings for rifampicin and ethambutol pharmacokinetic determinations

| **Autosampler and column oven (ACQUITY UPLC H-Class SM-FTN; Waters, Milford, MA, USA)** |
| --- |
| Purge solvent: 95/5 % (v/v) water/acetonitrile + 0.1% formic acid |
| Wash solvent: 80/20 % (v/v) water/methanol |
| Injection volume: 1 µL |
| Pre-inject wash time: 5 sec |
| Post-inject wash time: 5 sec |
| Needle placement: 4 mm |
| Column Temperature 25 ±1°C |
| Column: Acquity UPLC BEH C18 1.7 µm 2.1x100mm + assay frit 0.2 µm 2.1mm |
| Temp. tray: 10°C |

Table S11: Ionspray source used for rifampicin and ethambutol pharmacokinetic determinations

| **Ionspray source** |
| --- |
| Capillary: 2 kV |
| Polarity positive ion mode |
| Desolvation temperature: 500 °C |
| Desolvation gas flow: 950 L/h |
| Cone flow: 10 L/h |

Table S12: Mass spectrometer settings used for rifampicin and ethambutol pharmacokinetic determinations

| **MS settings (XEVO TQS-micro; Waters, Milford, MA, USA)** | | | | | |
| --- | --- | --- | --- | --- | --- |
| **Compound Name** | **Parent (m/z)** | **Daughter (m/z)** | **Dwell (s)** | **Cone (V)** | **Collision (V)** |
| Ethambutol | 205.0 | 116.0 | 0.05 | 26 | 14 |
| Ethambutol ^2^H_4_ | 209.2 | 120.0 | 0.05 | 26 | 14 |
| Rifampicine | 823.2 | 791.4 | 0.05 | 18 | 16 |
| Rifampicine ^2^H_8_ | 831.2 | 799.4 | 0.05 | 18 | 16 |
|  | | | | | |
| **Ionspray source** | | | | | |
| Capillary: 2 kV | | | | | |
| Polarity: positive ion mode | | | | | |
| Desolvation temperature: 500 °C | | | | | |
| Desolvation gas flow: 950 L/h | | | | | |
| Cone flow: 10 L/h | | | | | |

Accessories and disposables

Table S13: Accessories and disposable used for rifampicin and ethambutol pharmacokinetic determinations

| **Name** |
| --- |
| Finnpipette® Air displacement pipette 50-200 µL (ThermoFisher Scientific, Breda, The Netherlands) |
| Finnpipette® Air displacement pipette 5 - 40 µL (ThermoFisher Scientific, Breda, The Netherlands) |
| Finnpipette® Pipette tips 250 µL (ThermoFisher Scientific, Breda, The Netherlands) |
| HandyStep ® Repeater pipette (Brand, Wertheim, Germany) |
| HandyStep ® Repeater pipette tip 5 mL (Brand, Wertheim, Germany) |
| Autosampler vials: TPX micro-vial ND9 TPX clair 0.3mL 32x11.6mm (VWR, Amsterdam, The Netherlands) |
| Autosampler caps: PP Screwcap 9 mm pre-slit septum (VWR, Amsterdam, The Netherlands) |
| Safe-Lock tube 1.5 mL (Eppendorf, Nijmegen, The Netherlands) |
| DVX-2500 Multi-Tube Vortexer (VWR, Amsterdam, The Netherlands) |

Sample preparation

1.  Thaw and mix the (pre) created calibrators and qc

2.  In safe-lock tubes, dilute the calibrators and qc a factor 10 in RPMI 1640+2% FBS (e.g. 40 µL in 360 µL RPMI 1640+2% FBS)

3.  Mix the diluted calibrators and qc with a multi-tube vortex

4.  Pipette in a safe-lock tube: 50 µL sample, diluted qc or calibrator and add 150 µL PP solution

5.  Mix with a multi-tube vortex 20 sec. speed 2500 rpm

6.  Centrifuge 5 min (18.620 g)

7.   Add to the autosampler vials: 95 µL ascorbic acid 0.9 mg/mL and 75 µL supernatant

8.  Close the autosampler vial with a pre-slit cap

9.   Mix the autosampler vials with a multi-tube vortex 20 sec. speed 2500 rpm

10. Inject the sample(s) on the chromatographic separation system

Calculations

Acquired data was processed using Waters TargetLynx software (version 4.1). The calibration curves were linear plotted as log ratio signal response compound peak area and internal standard peak area versus log concentration.

Chromatogram


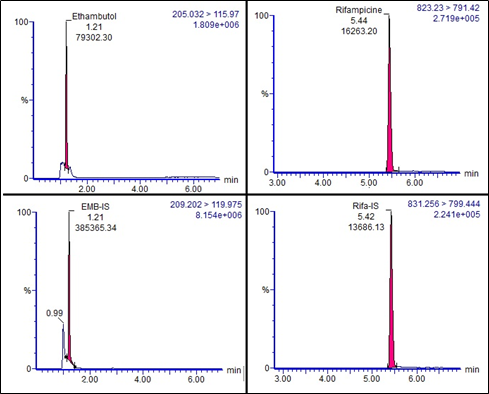


Figure S10: Chromatogram of the lowest calibrator with internal standards

Validation results

Table S14: Within – and between run accuracy and precision for ethambutol and rifampicin

|  | Conc. | Within run (n=5) | | Between run (n=15) | |
| --- | --- | --- | --- | --- | --- |
|  | (mg/L) | Accuracy (%) | Precision (%) | Accuracy (%) | Precision (%) |
| EMB | 0.0480 | 98.0 | 1.22 | 98.8 | 0.94 |
|  | 0.113 | 97.5 | 1.02 | 97.9 | 0.43 |
|  | 1.130 | 97.9 | 1.37 | 98.2 | 1.37 |
|  | 11.30 | 95.1 | 1.25 | 98.9 | 3.42 |
|  | 15.18 | 96.3 | 1.01 | 98.5 | 1.90 |
| RIF | 0.158 | 97.1 | 4.34 | 98.6 | 2.62 |
|  | 0.401 | 95.6 | 3.89 | 98.2 | 2.00 |
|  | 4.006 | 98.5 | 1.63 | 99.5 | 0.97 |
|  | 40.06 | 96.0 | 3.99 | 99.6 | 2.99 |
|  | 49.85 | 96.2 | 2.93 | 97.8 | 1.28 |

5.2.4.3. Clofazimine pharmacokinetic sample measurement protocol

Reference materials

- Clofazimine (Sigma-Aldrich, Zwijndrecht, The Netherlands)
- Clofazimine ^2^H_7_ (Alsachim, Illkirch-Graffenstaden, France)

 Chemicals and solvents

- Acetonitrile, Hypergrade for LC-MS (Merck, Darmstadt, Germany)
- Methanol, Absolute ULC/MS (Biosolve, Valkenswaard, The Netherlands)
- Dimethyl sulfoxide (Merck, Darmstadt, Germany)
- Formic acid, 98-100% for LC-MS (Merck, Darmstadt, Germany)
- Ammonium formate, Eluent additive for LC-MS (Sigma Aldrich, Zwijndrecht, The Netherlands)
- RPMI Medium 1640 (Thermo Fisher Scientific, Breda, The Netherlands)
- Fetal bovine serum (Thermo Fisher Scientific, Breda, The Netherlands)

Stock and work solutions

Three independent stock solutions (for calibrators, quality controls and a reserve) were prepared and stored at -40 °C. Clofazimine stock solution 1 mg/mL was prepared in DMSO.

The labelled internal standard stocks are prepared by dissolving the compounds in the same manner as the unlabelled compound stocks, only at a concentration of 1 mg/mL.

A protein precipitation solution containing 0.1 mg/L CFZ in acetonitrile/methanol 75/25 % (v/v) is prepared from the labelled internal stock solution. All stocks and solutions were stored at -40°C.

Calibration and quality control solutions

For the preparation of the calibration curve one of the stock solutions was used and diluted with methanol/water 50/50 % (v/v) to obtain seven calibration solutions 0.04, 0.125, 0.250, 0.625, 1.25, 5.00 mg/L CFZ.

Quality control samples are made at three levels (qc low, medium and high) from a different stock than the calibration solutions at 0.1125, 0.75, 3.75 mg/L CFZ.

All solutions were stored at −40 °C until analysis and are stable for at least 20 months.

Equipment and settings

Table S15: Pump settings used for ACQUITY UPLC H-Class FTN for clofazimine pharmacokinetic determinations

| **Pump settings (ACQUITY UPLC H-Class FTN; Waters, Milford, MA, USA)** | | | |
| --- | --- | --- | --- |
| **Time (min)** | **Flowrate (mL/min)** | **% A**  10mM ammonium formate buffer in water pH 4.5 | **% B**  10mM ammonium formate buffer in methanol pH 4.5 |
| 0 | 0.3 | 30 | 70 |
| 0.25 | 0.3 | 30 | 70 |
| 1.25 | 0.3 | 5 | 95 |
| 3.75 | 0.3 | 5 | 95 |
| 4 | 0.3 | 30 | 70 |
| 8.00 | 0.3 | 30 | 70 |
|  | | | |
| Seal wash: 90/10 % (v/v) water/acetonitrile | | | |
| Seal wash period: 5 min | | | |

Table S16: Autosampler and column oven settings for clofazimine pharmacokinetic determinations

| **Autosampler and column oven (ACQUITY UPLC H-Class SM-FTN; Waters, Milford, MA, USA)** |
| --- |
| Purge solvent: 95/5 % (v/v) water/acetonitrile + 0.1% formic acid |
| Wash solvent: 80/20 % (v/v) water/methanol |
| Injection volume: 1 µL |
| Pre-inject wash time: 5 sec |
| Post-inject wash time: 5 sec |
| Needle placement: 4 mm |
| Column Temperature 40 ±1°C |
| Column: Acquity UPLC BEH C18 1.7 µm 2.1x100mm + Frit 0.2 µm, 2.1mm |
| Temp. tray: 10°C |

Table S17: Ionspray source used for clofazimine pharmacokinetic determinations

| **Ionspray source** |
| --- |
| Capillary: 2 kV |
| Polarity positive ion mode |
| Desolvation temperature: 500 °C |
| Desolvation gas flow: 950 L/h |
| Cone flow: 10 L/h |

Table S18: Mass spectrometer settings used for clofazimine pharmacokinetic determinations

| **MS settings (XEVO TQS-micro; Waters, Milford, MA, USA)** | | | | | |
| --- | --- | --- | --- | --- | --- |
| **Compound Name** | **Parent (m/z)** | **Daughter (m/z)** | **Dwell (s)** | **Cone (V)** | **Collision (V)** |
| Clofazimine | 473.16 | 431.04 | 0.05 | 26 | 36 |
| Clofazimine ^2^H_6_ | 480.16 | 432.09 | 0.05 | 26 | 36 |
|  | | | | | |
| **Ionspray source** | | | | | |
| Capillary: 2 kV | | | | | |
| Polarity: positive ion mode | | | | | |
| Desolvation temperature: 500 °C | | | | | |
| Desolvation gas flow: 950 L/h | | | | | |
| Cone flow: 10 L/h | | | | | |

Accessories and disposables

Table S19: Accessories and disposable used for clofazimine pharmacokinetic determinations

| **Name** |
| --- |
| Finnpipette® Air displacement pipette 50 - 200 µL (ThermoFisher Scientific, Breda, The Netherlands) |
| Finnpipette® Air displacement pipette 5 - 40 µL (ThermoFisher Scientific, Breda, The Netherlands) |
| Finnpipette® Pipette tips 250 µL (ThermoFisher Scientific, Breda, The Netherlands) |
| HandyStep ® Repeater pipette (Brand, Wertheim, Germany) |
| HandyStep ® Repeater pipette tip 5 mL (Brand, Wertheim, Germany) |
| Autosampler vials: TPX micro-vial ND9 TPX clair 0.3mL 32x11.6mm (VWR, Amsterdam, The Netherlands) |
| Autosampler caps: PP Screwcap 9 mm pre-slit septum (VWR, Amsterdam, The Netherlands) |
| Safe-Lock tube 1.5 mL (Eppendorf, Nijmegen, The Netherlands) |
| DVX-2500 Multi-Tube Vortexer (VWR, Amsterdam, The Netherlands) |

Sample preparation

1.  Thaw and mix the (pre) created calibrators and qc

2.  In safe-lock tubes, dilute the calibrators and qc a factor 20 in RPMI 1640+2% FBS (e.g. 40 µL in 760 µLRPMI 1640+2% FBS)

3.  Mix the diluted calibrators and qc with a multi-tube vortex

4.  Pipette in a safe-lock tube: 50 µL sample, diluted qc or calibrator and add 150 µL PP solution

5.  Mix with a multi-tube vortex 20 sec. speed 2500 rpm

6.  Centrifuge 5 min (18.620 g)

7.   Add to the autosampler vials: 95 µL formic acid 1 mg/mL and 75 µL supernatant

8.  Close the autosampler vial with a pre-slit cap

9.   Mix the autosampler vials with a multi-tube vortex 20 sec. speed 2500 rpm

10. Inject the sample(s) on the chromatographic separation system

Calculations

Acquired data were processed using Waters TargetLynx software (version 4.1). Calibration curves were fitted linearly as the ratio of the peak area of the compound signal response and the peak area of the internal standard against concentration, and a weighting factor of 1/x2 was also used.

Chromatogram


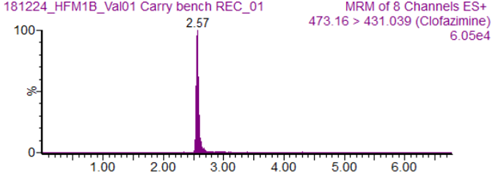


Figure S11: Chromatogram of the lowest calibrator with internal standards

Validation results

Table S20: Within – and between run accuracy and precision for clofazimine

|  | Conc. | Within run (n=5) | | Between run (n=15) | |
| --- | --- | --- | --- | --- | --- |
|  | (mg/L) | Accuracy (%) | Precision (%) | Accuracy (%) | Precision (%) |
| CFZ | 0.0407 | 108.78 | 5.45 6 | 103.50 | 3.98 |
|  | 0.1124 | 108.54 | 7.27 | 99.58 | 7.67 |
|  | 0.750 | 95.95 | 1.89 | 97.33 | 1.62 |
|  | 3.748 | 107.68 | 3.20 | 103.79 | 3.50 |
|  | 5.005 | 102.34 | 1.24 | 101.72 | 0.78 |

1. **Unexpected events during the experiment**

On day 5, there was power failure that did not affect the results of the experiment but caused flooding in some systems.

1. **References**

1. Banaschewski B, Verma D, Pennings LJ, Zimmerman M, Ye Q, Gadawa J, Dartois V, Ordway D, van Ingen J, Ufer S, Stapleton K, Hofmann T. 2019. Clofazimine inhalation suspension for the aerosol treatment of pulmonary nontuberculous mycobacterial infections. J Cyst Fibros 18:714–720.

2. Irwin SM, Gruppo V, Brooks E, Gilliland J, Scherman M, Reichlen MJ, Leistikow R, Kramnik I, Nuermberger EL, Voskuil MI, Lenaerts AJ. 2014. Limited activity of clofazimine as a single drug in a mouse model of tuberculosis exhibiting caseous necrotic granulomas. Antimicrob Agents Chemother 58:4026–4034.
